# Supplementary material for: Identifying and characterizing superspreaders of low-credibility content on Twitter
Source: PLoS One. 2024 May 22;19(5):e0302201. doi: 10.1371/journal.pone.0302201 (PMC11111090; doi:10.1371/journal.pone.0302201)
Supplement: Attachment — Submitted filename: response-letter.pdf [file pone.0302201.s001.pdf]

RE: PLOS ONE submission: PONE-D-23-36459

Dear Editor,

We would like to express our deepest appreciation for your guidance and the invaluable insights provided by the referees during the review of our manuscript. This detailed and constructive feedback has played a crucial role in enhancing the overall quality and clarity of our work. We have worked carefully to incorporate this feedback and firmly believe that the manuscript has greatly benefited from these revisions.

As requested, each comment is addressed individually in the pages that follow. However, for convenience, we enumerate below the central concerns that were raised, followed by an overview of how they are addressed.

1. Reframe the paper to be about "low-credibility content" instead of misinformation.
2. Clarify methodology aspects (add equations, motivate certain analyses, etc.).
3. Do not utilize the "FIB-index" terminology.

Overall, we have addressed these issues in the following ways. We altered the title and rewrote the entire manuscript to frame it as about "identifying and characterizing superspreaders of low-credibility content on Twitter." All notions of the FIB-index have been removed. Additionally, we have included equations for tested metrics and clarified the rationale for specific analyses.

More information is provided in the pages that follow, where we address each comment.

We hope that these revisions meet your expectations. In our latest submission, you will find two copies of the manuscript: one highlighting the changes made and another in its final, unmarked form.

Thank you for overseeing this rigorous review process and helping us improve our work.

Sincerely Yours,

Matthew R. DeVerna *et al.*
Observatory on Social Media
Indiana University Bloomington

1 Response to Referee 1

Summary

Reviewer 1 provided us with some great insights. We have attempted to capture nearly all of these thoughts within the manuscript, in one way or another, and provide clarity in some areas of confusion.

One key point of concern was related to our use of language related to "misinformation" as we had previously operationalized misinformation based on low-credibility sources. To address this issue, we have rewritren the paper to focus explicitly on low-credibility content. The title has been altered, as well as nearly all mentions of the word "misinformation," unless we were specifically referring to the topic more generally or other research.

It was suggested that our use of the term "superspreaders" may create some confusion within the field of epidemiology. With respect to this issue, the reviewer also referenced work by the Election Integrity Partnership (EIP). We address this issue in three distinct ways detailed in our response to Comment 1.3. First, we define and clarify our operationalization throughout the paper. Second, we highlight this limitation in the revised *Discussion* section, highlighting it as an important area of future research. Third, we discuss the work of the EIP in detail to clarify some important distinctions.

Finally, confidence intervals and effect sizes were included for statistical comparisons.

Comment 1.1

Every review should open with a bit of praise, and there's plenty to work on for dolling that out here. The authors have compiled a unique data, one that we probably won't see recreated again anytime in the next several years. They work through a careful and quantitative analysis, asking clear questions and explaining both their goals and methods in a clear manner. This paper should be published in PLoS ONE and I hope it is. However, before I can accept the manuscript I think it needs a pretty serious makeover in terms of its framing. Not only do I think this will make the paper more "correct", I think it will help make it a more valuable piece because it will provide deeper insight into a specific phenomenon and not get washed away in the flurry of pieces about superspreaders and misinformation. I'll review that concern in the overview, my thinking behind it, and provide some examples of why I believe the current framing may be misleading. By all means, the authors should feel free to push back but hopefully they'll take this to heart. I get that there's a lot below, but the quantity is a reflection of what I think the paper can do, more than what it didn't do!

Response:
We sincerely thank the referee for their kind words about our work and for the effort in providing detailed feedback and suggestions. As you will see, we agree with the vast majority of their feedback and work to incorporate it where possible. In other cases, we feel there may be some confusion resulting from a lack of clarity in our writing and attempt to remedy this issue with careful revisions.

Comment 1.2

The authors operationalize misinformation superspreaders based on various network features, their propensity to share misleading content, and how far the misleading content they share/amplify tends to travel. While low-quality sources are likely to be misleading, I don't think this really encapsulates the variety of misinformation shared during the pandemic and the strategies by which it was spread.

For instance, an article in the BMJ amping up an ostensible Pfizer whistleblower was the most widely shared scientific article in the pandemic with 78330 shares via altmetrics. Interestingly, the originator of this intentionally misleading piece, Paul Thacker, is cited in the present manuscript as ref 46. Thacker has been responsible for quite a bit of anti-vaccine misinformation, either as a source or resharing.

More generally, scientific articles were often weaponized, as were text-based threads, images, and videos shared on platform. The University of Washintgon's IHME models fit pandemic dynamics to a gaussian function and led to predictions that the "wave would pass" and the pandemic would be over within months. Several faculty at Stanford early on produced academic research arguing that no more than 10,000 Americans would die and stood by that claim even when it was shown unequivocally to be false by some of the worlds' top statisticians. Similarly VAERS data, reports of random death, etc... was weaponized repeatedly by anti-vaccine communities to create misleading narratives about vaccines. Even the WHO maintained that covid was not airborne for two years, against scientific consensus. This isn't peculiar to pandemic-related misinformation, but also applied heavily during the election as well. Some accounts simply used text and made misleading statements, others relied on images, links to content on youtube (this was big), etc... and would not be swept up in a definition based on sites ranked low in quality.

There's no reason to believe that misinformation super-spreaders all use the same strategy, particularly across the political spectrum or various ideological divides that emerged during covid. My first concern is that the write up is about super-spreaders of misinformation but it's really getting at super-sharers-of-low-quality-websites. I think the paper should be reframed to perhaps frame this as super-spreaders of low-quality information rather than misinformation.

Response:

The reviewer has brought up several key points, and we concur with their reasoning. Consequently, we have revised the manuscript to refer to "superspreaders of low-credibility content" and operationalized this definition based on low-credibility sources. For a detailed overview of the changes, we invite you to consult the track-changed version of the paper, as the modifications are too extensive to list individually. Additionally, we have removed the earlier mentioned reference, as it is not critical to our study's core message.

Comment 1.3

A related issue is that, as far as I can tell, retweets are linked to the original source, not to the person who caused the retweet to be in an individuals' feed. I think this is a bit of an

issue, because often super-spreaders amplify misinformation rather than generate it. The EIP report details this phenomenon at length. In disease, superspreaders would typically not refer just to index cases, as here, but to anyone whose individual reproductive number is high (i.e., anyone in the transmission chain who forwards it along to many others). As such, I worry that the superspreader definition in the sense of Lloyd-smith 2005 might be quite different from how its described here. There should be some discussion clarifying this difference and the write-up should caution that these are influential original sources of low-quality link sharing rather than super-spreaders of misinformation.

The reason this is more than pedantic is that some of the most influential accounts wield that power via retweets of smaller sized accounts. Our understanding of super-spreading here actually presents quite a bit of conflict with the operationalization in the study. We know, from empirical work, theory and simulations that large accounts that retweet are responsible for a substantial amount of spread and their influence here gets foisted upon the person they tend to retweet. If you were tasked with removing an account, basing it on the definition in the paper would have a smaller impact on the spread of misinformation than if we had information about account amplification. For example, let's say there's an account that is smaller and repeatedly posts misinformation that is amplified reliably by an account that follows them with 100M followers. FIB-index and influence here would suggest removing the smaller account would reduce spread of misleading links. Upon doing so, the 100M follower account simply amplifies a different account also sharing misleading links.

I think all of the above are very real and insurmountable changes for framing this paper as a study of super-spreading of misinformation. That said, they present no problems to a study on influential originators of information from low-quality websites.

The main thing I would like to see in the rewrite is to be a bit more cautious about definitions and write this up as what it is—a clear study on the influential originators of widely shared links to misleading websites. I think that is quite different from super-spreaders of misinformation, but nonetheless an interesting and important contribution to the literature. I understand this is less catchy, but I think the study has either the option of being a fairly flawed study of superspreading of misinformation or a very good study who influentially shares links to low-quality websites. I am sure there is overlap in these populations, but I worry that we're missing most of the iceberg here.

Response:

The reviewer is correct about this insightful observation and indeed we are fully cognizant of the issue of amplification vs. origination of a post. Since the analysis in this paper is based on the cascade trees as returned in Twitter's raw data ("stars"), we have revised the text to explicitly define "superspreaders" as accounts that "introduce untrustworthy sources, which then disseminate widely." Specifically, we include the following language:

> *To answer our first research question, we begin by collecting 10 months of Twitter data and defining "superspreaders" as* ***accounts that introduce low-credibility content****, which then disseminates widely.*
>
> – Introduction (lines 49–51)

This point is also clearly stated in the section of the manuscript that explains our dismantling

analysis. See, for example, the below excerpts:

> *We create four separate rankings of the 47,012* ***users that created at least one post containing low-credibility content*** *during the observation period based on each of the metrics defined above...*
>
> – Accounting for future low-credibility content (lines 199–201)

> *As we remove account i from the network, we also remove all retweets of posts containing low-credibility content* ***originated*** *by i, i.e., the outgoing edges from i.*
>
> – Accounting for future low-credibility content (lines 204–205)

Furthermore, we have added a new paragraph in the *Discussion* section that highlights the role of potential amplifiers as a fruitful role for future research:

> *The current work is specifically focused on original posters of low-credibility content and their disproportionate impact. However, it opens the door for future research to delve into the roles of "amplifier" accounts that may reshare misinformation originally posted by others (Kennedy et al., 2022).*
>
> – Discussion (lines 470–473)

Properly defined in this way, we believe the term "superspreader" remains apt for continued use, given its recognition in both the misinformation literature — "*Given the high volume of posts shared or consumed by superspreaders of fake news...*" (Grinberg et al., 2019) — and popular media (Frenkel, 2020). This term, familiar to our audience, will connect with established discussions, while our precise definition ensures accurate representation in the context of our study. We hope this approach strikes a balance between familiarity and precision.

Still, we appreciate the important distinction between the two potential roles of a "superspreader" (originator vs. amplifier), and, in fact, we are currently exploring this distinction in our lab. Teasing apart the two potential roles requires reconstruction of the retweet cascades, which is a challenging inferential problem on its own. While this falls outside the scope of the present paper, we would like to share the results of some preliminary analyses with the reviewer to assuage their concern about our use of the term "superspreaders."

In a related project, we examine whether amplifiers significantly contribute to spreading low-credibility content by attempting to reconstruct retweet cascades (using certain heuristics) to infer the true diffusion of information. This procedure allows us to construct two versions of the same retweet network. A "naive" version is built in the typical way, from the star-based Twitter data, while a "reconstructed" version utilizes the reconstructed cascades. We then dismantle the reconstructed network in two ways, prioritizing nodes to be removed based on their degree in the naive vs. the reconstructed network. If amplifiers were more influential than originators, the dismantling would be quicker based on the node degree in the reconstructed network. To our surprise, we are finding that, while some accounts *do* amplify cascades they did not originate, when aggregating across cascades it is more efficient to dismantle based on the node degrees in the naive network. This is the case both because many amplifiers are also originators, and because influential originators are highly active, often tweeting constantly every day. These preliminary findings suggest

that originators are overall more influential than amplifiers. We plan to report on these results in a separate paper presenting a new class of heuristic reconstruction algorithms.

We recognize that, upon initial review, there may be an apparent discrepancy between our findings and those of the Election Integrity Partnership (EIP) study mentioned in the reviewer's comments. However, it's important to emphasize that the units of study used in the two analyses are different: we analyze amplifiers' influence on the spread of low-credibility content by focusing on a user's role in individual retweet cascades, while Kennedy et al. (2022) focus on aggregated "stories" (or "narratives"). The latter approach aggregates multiple related retweet cascades under one story. Furthermore, the use of *originators* of low-credibility content seems to align with the method used by the EIP:

> *In Figure 3B, we only count stories [...] where an account contributed* ***original content that was highly retweeted*** *as part of a misinformation story. This is also the metric we use for our repeat spreaders analysis below.*
>
> *– Kennedy et al. (2022), pg. 23*

Our findings align as well, in terms of the disproportionate influence of a small number of accounts that disseminate unreliable content.

In conclusion, we believe that the initial findings from our related project are the first to empirically delve into the complexities that the reviewer has highlighted related to the different roles of originators and amplifiers. While this work merits its own publication, we are hopeful that these preliminary findings — which indicate that amplifiers may not be as pivotal in spreading content as we may have assumed — along with the revised language in our manuscript, will assuage the reviewer's concerns.

Comment 1.4

The dismantling analysis is interesting, and it's clear that it demonstrates a consistency between the observation and evaluation period (i.e., people who share sources from low quality sites tend to continue to do so). At the same time, it's not clear the extent to which the relative performance of the metric merely reflects tautology between the definition of the retweet network and the definition of the metric. While I understand the analysis, it's unclear what it's telling us beyond that the retweet network is pretty stable, and that h-index and Influential both measure retweets within the retweet network. Perhaps there's a less convoluted way to show this?

Response:
Thank you for your feedback. We too had this concern early on in our project, when we conducted the dismantling analysis on the same retweet network used to rank the accounts. The results in this case could in fact be attributed to a tautology or overfitting, similar to when a machine learning algorithms is evaluated on the test set. It is precisely to avoid this pitfall that we decided to do the evaluation in a different period, so that we could assess the predictive power of the ranking methods.

As the reviewer observes, our analysis does reveal that the retweet network exhibits a degree of

"stability." However, it's important to emphasize that the prediction results are not a given; rather, they form one of the central discoveries of our research. Specifically, we demonstrate that the retweet network's (topological) consistency over several months can be leveraged to calculate simple metrics (h-index, total retweet count) that serve as reliable *predictors* for identifying superspreaders of low-credibility content. If the retweet network lacked the (topological) stability we identified, these metrics would yield poor results and the prediction task would be significantly more challenging. That being said, it is important to note that not all the features of a retweet network may be stable over time. For instance, if account $\mathcal{I}$ is identified as an influential account during time period $\mathcal{T}$, while $\mathcal{I}$ may continue to be influential during period $\mathcal{T}+\delta\mathcal{T}$, the accounts that are retweeting $\mathcal{I}$ need not be the same since the metrics we have used are oblivious to this. In this way, the actual features of the retweet network may differ with time.

To address the editor's feedback, we left the dismantling analysis intact but clarified its motivation within the *Accounting for future misinformation* section:

> *This work seeks to predict which Twitter accounts will be superspreaders of untrustworthy content in the future. To this end, we identify accounts in the observation period and then quantify how much low-credibility content they spread during the evaluation period.*
>
> – Accounting for future low-credibility content (lines 190–193)

Comment 1.5

For the political leaning bit, I think this is somewhere that the difference between misinformation and source quality really kicks in. Several very prominent left-leaning accounts shared substantial misinformation about the pandemic, but often made their case by exaggerating news from reliable sites. As terrible as the pandemic was, it wasn't–for instance–airborne HIV or various other narratives that took off.

Response:
Thank you for your feedback. This concern should be addressed by our revisions of the language of the paper to refer more clearly to low-credibility sources rather than misinformation.

Comment 1.6

Throughout: Estimates need bounds placed on their uncertainty (e.g., confidence intervals, credible regions etc. . .) wherever possible. Somewhat relatedly, I'd be careful about inferences drawn from the presence/absence of statistical significance in various places. Ideally anywhere that has an estimate or a p-value should have an interpretable effect size (e.g., not just a test statistic like U) with quantified uncertainty.

Response:
Thank you for your feedback. We have accompanied all p-values with Cohen's d effect sizes and 95% confidence intervals, calculated using a bootstrapping method.

Comment 1.7

Minor Points: L11: The second paragraph of the introduction is just a single sentence, consider folding it into P1 or P2.

Response:
Thank you for your feedback. We have merged this paragraph with the one that follows.

Comment 1.8

I urge you just to call the FIB-index the h-index. No point in renaming it again if it's the same metric! Other research relying on it outside of its original content typically uses the same term and this will be more searchable/clearer to the reader.

Response:
Thank you for your feedback. We have revised the manuscript to use "h-index" in place of "FIB-index."

2 Response to Referee 2

Summary

Reviewer 2 also provided insightful feedback, particularly regarding the related work and the need for enhanced clarity and context in sections affected by Twitter's transition to X. In response, we have thoroughly revised our manuscript, integrating these suggestions to address the concerns raised.

Comment 2.1

The manuscript "Identification and characterization of misinformation superspreaders on Twitter" by Matthew R. DeVerna et al. presents a study of misinformation superspreaders on Twitter and proposes a method to predict which accounts will become superspreaders. The work is interesting and relevant. The methodology is sound. I have only minors points that the authors can easily address. Apart from this, I recommend its acceptance.

Response:
We are grateful to the reviewer for their encouraging comments and the recommendation for our article to be accepted for publication. In the following responses, we earnestly address and aim to resolve the concerns that have been highlighted.

Comment 2.2

The main point to address is the fact that Twitter does not really exists anymore, or at least not in the same form than it was during this study. I think the proposed method can be generalized to other platforms, so this is not a crucial issue, but the authors only used Twitter data, so they should still address this point in the introduction and discussion.

Response:
Thank you for highlighting this important point. We speak to this within the revised *Discussion* section:

> *This study relies on data obtained prior to Twitter's transformation into X. At that time, Twitter was actively experimenting with ways to mitigate the spread of misinformation (Collins, 2020). This is starkly contrasted by X's recent decisions to lay off much of their content moderation staff and disband their election integrity team (Collins et al., 2022; Elliott and Stokel-Walker, 2022). Despite these changes, the key mechanism studied here —a user's ability to reshare content— remains a fundamental aspect of the platform.*
>
> – Discussion (lines 474–479)

Comment 2.3

The second point is that I think the authors could refer to the results of two other studies that have looked closely at Twitter misinformation superspreaders

- Bovet, A., & Makse, H. A. (2019). Influence of fake news in Twitter during the 2016 US presidential election. Nature Communications, 10(1), 7. [source]
- Flamino, J., Galeazzi, A., Feldman, S., Macy, M. W., Cross, B., Zhou, Z., Serafino, M., Bovet, A., Makse, H. A., & Szymanski, B. K. (2023). Political polarization of news media and influencers on Twitter in the 2016 and 2020 US presidential elections. Nature Human Behaviour, 7(6), 904–916. [source]

These studies report the fraction of verified/unverified/deleted, politically or media affiliated top misinformation superspreaders in 2016 and 2020, so it seems very natural to summarize them in the related work and compare what the authors found with them.

Response:
Thank you for bringing these references to our attention. We now discuss this work within the *Related work* section:

> *Another approach that has been applied in social media studies (Flamino et al., 2023; Bovet and Makse, 2019) treats this task as a problem of optimal percolation in random networks (Morone and Makse, 2015). The goal is to minimize the energy in a many-body system to pinpoint the smallest possible set of influential accounts (Morone and Makse, 2015).*
>
> – Related work (lines 103–106)

Comment 2.4

The third point is about the account classification (Fig. 2). 128 acounts were classified as not active anymore, but the authors should still look at what kind of accounts they were and included them in the other categories (media, political, ...).

Response:
Thank you for this suggestion. Regrettably, the suspension of the accounts in question means their profiles are no longer accessible, which prevents us from annotating those accounts and performing the suggested analysis. We appreciate your understanding of this constraint.

Comment 2.5

Another point that the authors should comment on is how the fact that Twitter was actively combating misinformation in 2020 could impact their result.

Response:
Thank you for bringing this to our attention. We now mention this in the revised *Discussion* section. Please see the above response to Comment 2.2 for this language.

Comment 2.6

A small detail: it's not clear in the text [if] r_m is the "ratio of tweets linking to low-credibility content" (line 239) or if it is the "proportion of shared sources linking to low-credibility content" (line 252), i.e. if the same source is counted multiple times or not.

Response:

Thank you for bringing this to our attention so that we may clarify. Our intention here is to measure the degree to which a user shares low-credibility content when they share a link on the platform. Thus, for an individual user we are calculating the proportion of tweets linking to any low-credibility source, including multiple stories from a single untrustworthy outlet and even multiple tweets linking to the same story, out of all tweets linking to any source. We clarify this point by including an explicit equation in the *Source-sharing behavior* section and altering the language to remove ambiguity.

> *Specifically, for a given account, we calculate the ratio* $r_m = \frac{|\mathcal{T}_i|}{|\mathcal{P}_i|}$, *where* $|\mathcal{T}_i|$ *represents the total count of user i's posts that link to low-credibility sources and* $|\mathcal{P}_i|$ *is the count of all posts by user i that link to any source during the observed period.*
>
> – Source-sharing behavior (lines 247–249)

References

[1] Alexandre Bovet and Hernán A Makse. “Influence of fake news in Twitter during the 2016 US presidential election”. In: *Nature Communications* (2019).

[2] Ben Collins. “Twitter is testing new ways to fight misinformation — including a community-based points system”. In: *NBC News* (2020).

[3] Ben Collins et al. “Twitter lays off employees who fight misinformation”. In: *NBC News* (2022).

[4] Vittoria Elliott and Chris Stokel-Walker. “Twitter’s Moderation System Is in Tatters”. In: *WIRED* (2022).

[5] James Flamino et al. “Political polarization of news media and influencers on Twitter in the 2016 and 2020 US presidential elections”. In: *Nat Hum Behav* 7 (2023), pp. 904–916.

[6] Sheera Frenkel. “How Misinformation ‘Superspreaders’ Seed False Election Theories”. In: *The New York Times* (2020).

[7] Nir Grinberg et al. “Fake news on Twitter during the 2016 U.S. presidential election”. In: *Science* (2019).

[8] Ian Kennedy et al. “Repeat Spreaders and Election Delegitimization: A Comprehensive Dataset of Misinformation Tweets from the 2020 U.S. Election”. In: *Journal of Quantitative Description: Digital Media* (2022).

[9] Flaviano Morone and Hernán A. Makse. “Influence maximization in complex networks through optimal percolation”. In: *Nature* 524 (2015), pp. 65–68.
